# Nutrition assessment and geriatric associated conditions among community dwelling Iranian elderly people

**DOI:** 10.1186/s12877-020-01668-8

**Published:** 2020-08-06

**Authors:** Afsaneh Bakhtiari, Mahbobeh pourali, Shabnam Omidvar

**Affiliations:** 1 Social Determinants of Health Research Center, Health Research Institute, Babol University of Medical Sciences, Babol, I.R. Iran; 2 Department of Nursing, Babol School of Midwifery Nursing, Babol University of Medical Sciences, Babol, Iran; 3 Social Determinants of Health Research Center, Health Research Institute, Babol University of Medical Sciences, Babol, I.R. Iran

**Keywords:** Nutritional screening, Malnutrition, Elderly, MNA

## Abstract

**Background:**

Although malnutrition risk is well documented in elderly care institutions, few studies have been conducted to address concerns regarding community-dwelling elderly people. This study has been aimed to describe the nutritional status and its related socioeconomic and geriatric factors in community-dwelling elders with malnutrition.

**Method:**

For this study, a randomized sampling among people aged 60 has been done (*n* = 326). Information on nutrition status (full MNA) and health information, like cognitive status (MMSE), daily functional status (ADL and IADL scales) and frailty was obtained. Multiple logistic regression analyses have been carried out, in order to identify the association of demographical and clinical factors with malnutrition.

**Results:**

28.1% of the participants suffered from poor nutrition. In the binary analysis, low MNA scores were associated with increasing age, female gender, lower education level, financial dependence, solitary life, poor self-rated health, multiple physical disabilities and chronic disease, polypharmacy, smoking, functional and cognitive decrease and frailty. In the final model of the multivariate analysis, living alone (OR:1.249,CI:1.105–2.620), multiple physical disabilities (OR:2.183,CI:1.246 ± 3..250) and chronic disease (OR: 2.148,CI:1.167–2.879) were independently associated with malnutrition. Also financial independency (OR:0.625,CI:0.233–0.938), functional ability on ADL (OR:0.536,CI:0.327–0.976) and IADL (OR:0.319,CI:0.194–0.856), normal cognitive (OR:0.456,CI:0.293–0.934) and no frailty (OR:0.253,CI:0.117–0.729) independently were inversely associated with malnutrition. The model was adjusted for all socio- demographic and health variables that were significantly related in the previous models.

**Conclusions:**

Our results indicated a strong correlation between malnutrition and health status. Identifying predictive factors can potentially improve prevention and management strategies used for malnutrition in elderly.

## Background

Malnutrition in the elderly is defined as undernourishment, described by inadequate food intake, poor appetite, and loss of muscle and weight [1]. Health status and life quality are heavily affected by malnutrition [2]. Dependence, solitude and chronic diseases are such factors that affect it [3]. Malnutrition in the elderly is associated with a high social burden that encompasses a multi-dimensional concept including physical and psychological aspects [1].

The prevalence of malnutrition in the elderly living in the community is relatively low (6–11%); however the rates are higher among hospitalized people or those in residential care centers (32–64%) [4, 5]. Estimates show that 42% of the elderly residents of institutions and 51% of hospitalized elderly patients in the United States are affected by malnutrition [6]. In Europe and Asia [3, 7, 8] prevalence of malnutrition varies from 12 to 84%. The prevalence of malnutrition is estimated to be 9.2% among elderly living at home and 21.6% among elderly residents of nursing homes in Iran [7].

This variation depends on the malnutrition diagnostic criteria, the country investigated, the population being rural or urban, residence type, having multiple chronic diseases, and the level of socioeconomic status. Despite these remarkable data, nutritional problems are still not recognized as a necessity in the management of the elderly. Evaluation and nutritional interventions are very important in this population with high prevalence of chronic diseases including depression, dementia, functional disorders, polypharmacy [9, 10]. Malnutrition in elderly can lead to multiple health concerns, including weak immune system, increased risk of infections, muscle weakness and bone-loss followed by an increase in falls and higher risks of hospitalization and death [11, 12].

Despite the fact that previous studies have investigated determinants of malnutrition, research has not focused on a possible joint effect of multiple health factors that contribute to poor nutritional status among Iranian population. In addition to that, much of the previous researches on screening and prevention has largely been focused on the disabled elderly or resident in institutions; so the healthy elderly people were somewhat ignored. This study is aimed to determine the prevalence of malnutrition in community-dwelling elderly. The relationship between socio-demographic characteristics, as well as physical and mental health indicators and nutritional status has also been evaluated.

## Methods

### Design and study population

The participants were 331 community dwelling elderly, aged 60 years and over, living in Babol city, from Mazandaran province, Iran. Babol is a northern City of Iran with 12 urban health care centers out of which, 6 centers have been selected randomly.

Convenience sampling technique was used to choose among the elderly people attending the areas, serviced by each responsible health center, including elderly entertainment venues, parks and mosques. Considering these factors, 55 elderly citizens entered out study from each health center.

Eligible elderly were not diagnosed with Alzheimer, intense mental disorders or serious diseases. Individuals would opt out from the study for the absence of consent or incomplete filled out questionnaire. Hence, 326 questionnaires were analyzed out of a total of 331 questionnaires. The questionnaires were completed by participants in the presence of the researcher (for illiterate individuals, the researcher filled out the questionnaire on their behalf).

### Measurements and tools

A standard multi-part questionnaire was used to assess nutritional status as an outcome variable and some other explanatory variables.

### Nutritional status

Assessment of nutritional status of the subjects was conducted by a Persian version of the mini nutritional assessment (MNA) [13]. The MNA is the best tool which is validated, established and is a widespread tool for assessing nutritional status of elderly people [14]. The tool includes 18 questions regarding anthropometric, general, dietetic and subjective assessment. The questionnaire total score ranges from zero to thirty. Less than 17 points indicate malnutrition; 17 to 23.5 signify the risk of malnutrition, while equal or above 24 show a normal nutritional status. In our study, malnutrition status was defined as the risk of malnutrition or malnutrition.

### Socio-demographic variables

The Socio-demographic variables are composed of gender, age, place of living, marital status, education, number of children, occupation, residential home type, financial dependency, and adequacy of family income. All participants were covered by health insurance.

### Health status

Health status was described by chronic illnesses, type and amount of medications taken daily; as prescribed by a physician. We also looked for physical disabilities (including eyesight, hearing, dental, motion and talk disorders), psychological problems (stress, insomnia), smoking status and self-rated health.

Functional ability was analyzed through Katz Index of Independence in Activities of Daily Living (ADL) and Lawton –Brody Instrumental Activities of Daily Living Scale (IADL) [15, 16]. ADL is the most appropriate tool to determine functional status by analyzing the client’s ability to perform daily activities, independently. This tool is typically used by clinicians to detect functioning problems in performing daily activities and to provide appropriate care. According to the index, performance adequacy is ranked in six function categories of bathing, toileting, dressing, transferring, continence, and feeding. Clients’ answers for independence are recorded as yes/no in each of the six functions categories. Full function is indicated by a score of 5 and higher.

IADL is an appropriate tool to analyze independent living skills. The skills that are assessed in this tool are more complex compared to the basic daily activities, as measured by Katz Index of ADLs. The instrument is most useful for identifying the way a person functions at the present time, and to identify any improvement or deterioration over time. There are eight areas of performance, measured by the Lawton IADL scale. Women’s performance is rated, in all eight areas. The areas of food preparation, housekeeping, laundering have been historically excluded for men. Clients are scored in each category according to their highest level of performance. For women a summary score ranges from 0, indicating dependence and low function, up to 8, which shows high function and independence. For men, the score ranges from 0 to 5. Full function is indicated by the scores of more than 7 and 4 for women and men respectively. Moderate impairment in women and men is shown by a score of 4–6 and 2–3, and severe functional impairment by the score of 3 and 1 or less, respectively.

Cognitive status was described by the mini-mental state examination (MMSE). Since the cut-off point of 23 was reported for this test in the Iranian population [17], the results were grouped into low cognition (score ≤ 23) and normal cognition (score ≥ 24).

Frail status of the participants was assessed based on the five criteria by FRAIL scale items in AAH [18]. The FRAIL scale includes five components: Fatigue, Resistance, Ambulation, Illness, and Loss of weight. Frail scale scores range from 0 to 5 (i.e., 1 point for each component; 0 = best to 5 = worst) and represent frail (3–5), pre-frail (1–2), and robust (0) health status. Fatigue was measured by asking respondents how much time during the past 4 weeks they felt tired with responses of “all of the time” or “most of the time” scored 1 point. Resistance was assessed by asking participants if they had any difficulty walking up to 10 steps independently without resting or assistance. Ambulation was also assessed by the ability of walking alone for a hundred yards without any aids; a score of 1 is given to “yes” responses. Illness was scored 1 for respondents who reported five or more illnesses out of 11 total illnesses. Loss of weight was scored 1 for respondents with a self-reported weight decline of 5% or greater within the past 12 months. The associations of FRAIL scale scores categorized as frail or pre-frail (versus healthy) were examined with poor outcomes.

### Ethics approval

Ethics standards on human experimentation, as stated by the responsible institutional and national committee and the Declaration of Helsinki, were properly followed while carrying out procedures. Moreover, the study was approved by the ethics committee of Babol University of Medical Sciences (NO.:MUBABOL.REC.1388.1). All participants provided an informed written and signed consent form. All patients provided written informed consent.

### Analysis

Data were analyzed using the SPSS version 19.0. X^2^ test was used to assess binary associations between socio-demographic variables and physical and mental health indicators and nutritional status. ANOVA test was also applied to compare means of numerical variables including age, number of children and physical disabilities with nutritional status classes. In addition to those, multiple logistic regression was used to determine independent variables related to nutritional status so that all independent variables were entered simultaneously in a full model. Nutrition status was considered as the dependent variable in two classes of poor (malnourished or at risk) versus normal. The independent variables that were associated with nutritional status in the binary analysis (*p* ≤ 0.05) were included in these models. In Model 1, all socio-demographic predictors of nutritional status were entered at the same time. Model 2 presented health-related features (physical disabilities number, daily drug intake, self-rated healthy, chronic disease number, smoking status, MMSE, ADL, IADL, frailty). All variables that were significantly associated in the previous two models as independent variables were included in Model 3.

## Results

### Participants’ nutritional status

The study sample consisted of 115 elderly women and 211 men. Their mean age was 68.82 ± 7.15 years with the prevalence of malnutrition and risk of malnutrition 3.0 and 25.1%, respectively. Women’s nutritional status was considerably worse than men.

### Nutritional status and its related factors

Personal characteristics` binary analyses are depicted on Table 1. Individuals with higher age, female gender, more children, less income and life without spouse (living alone or with children) had poorer nutritional status (*p* < 0.001). 46% of the participants who lived with their children were either malnourished or at risk of malnutrition, while it was lower in the elderly who lived alone (33.4%). Greater level of nutritional disorders was also observed amongst illiterate people (46%).

**Table 1:** Associations between personal characteristics and nutritional status in elderly people

Variables	N	Malnutrition %	At malnutrition risk %	Normal-nourished %	***p***
**Age** Mean (SD)	326	70.00 ± 7.28	70.81 ± 7.43	68.21 ± 6.95	0.015
**Age group (y)**	326				0.019
60–75	259	3.1	22	74.9	
≥ 76	67	3	38.8	58.2	
**Gender**	326				0.001
Female	115	4.5	34.1	61.4	
Male	211	2.8	21.7	75.5	
**Marital status**	326				0.015
Married	236	3	21.2	75.8	
widowed/divorced/single	90	3.3	36.7	60	
**Education**	326				0.001
Illiterate	103	5.2	40.8	54	
Primary/middle school	77	4.9	24.7	70.4	
high school	89	1.1	12.4	86.5	
University	57	–	19.3	80.7	
**Residency**	326				0.084
Urban	269	2.6	23.4	74	
Rural	57	5.3	35.1	56.9	
**Financial dependency**	323				0.001
Dependent	41	7.5	30	62.5	
Independent^1^	282	2.1	21.4	76.5	
**Family Income**^2^	324				0.001
Inadequate	107	6.6	36.8	56.6	
Almost enough	121	5.6	28.5	65.9	
Adequate	96	–	13.5	86.5	
**Occupation**	326				0.223
Unemployed	233	2.6	28.3	69.1	
Part-time work	51	5.9	21.6	72.5	
Full-time work	42	2.4	14.3	83.3	
**The house**	326				0.629
Rent	13	–	30.8	69.2	
children home	5	–	–	100	
own	308	3.2	25.6	71.1	
**Live with**	326				0.001
Alone	51	2	31.4	66.7	
Spouse	236	2.9	21.6	75.4	
Children	39	5.1	41	53.8	
**Children number** Mean (SD)	325	5.10 ± 0.87	4.45 ± 1.98	3.61 ± 1.74)	0.001

^1^Including Employed, Pension, Rental property

^2^Self-reported

Health status is shown in Table 2. Malnutrition is considerably more common in individuals with multiple physical disabilities (including eyesight, hearing, dental, motion, talk disorders) and comorbidity (coexistence of chronic diseases) and poor self-rated health (*p* < 0.001). In regards to functional capacity (ADL and IADL), the findings showed functional decline in malnourished individuals and those at malnutrition risk compared to individuals with good nutritional status (*p* < 0.001). In addition to that, the largest proportions of frail subjects were at risk of malnutrition. Risk of malnutrition was found in 8.1% of the non-frail, compared to 37.3% in the pre-frail and 58.3% in the frail participants (*p* < 0.001). Furthermore, an association between cognitive function and nutritional status was demonstrated (*p* < 0.001). Normal cognitive performance is associated with decreased ratios of malnutrition and at risk individuals.

**Table 2:** Associations between health status and nutritional status in elderly people

Variables	N	Malnutrition %	At malnutrition risk %	Normal-nourished %	***P***
**Physical disabilities number**^**1**^ mean (SD)	318	2.50 ± 1.08	2.34 ± 0.92	1.82 ± 0.83	0.001
**Daily drug intake**	326				0.001
≤ 3 drugs	149	2	11.4	86.6	
>4 drugs	176	4	37.5	58.5	
**Self-rated healthy**	326				0.001
not healthy	53	9.4	62.3	28.3	
not sure	37	5.4	40.5	54.1	
Like others	122	1.6	21.3	77	
Better than others	114	0.9	7.9	91.2	
**Chronic disease number**	322				0.001
< Three disease	224	3.1	19.8	77.1	
≥ Three disease	98	10.8	36.4	52.8	
**Psychological problem**	325				0.251
None	14	–	7.1	92.9	
Stress	241	2.1	17.8	80.1	
insomnia	70	5.7	52.9	41.4	
**Smoking status**	326				0.001
Never	292	2.7	17.6	79.7	
Smoker	34	5.9	26.4	67.7	
**MMSE**	322				0.001
Low cognition	54	9.26	46.30	44.44	
Normal cognition	268	1.87	20.52	77.61	
**ADL**	324				0.001
Dependent	42	11.9	57.1	31	
Independent	282	1.8	20.9	77.3	
**IADL**	325				0.001
Dependent	28	14.3	60.7	25	
Moderate	67	6	47.8	46.3	
Independent	230	0.9	14.3	84.8	
**Frailty**	328				0.001
No	151	1.3	8.1	90.6	
Pre frail	153	4	37.3	58.7	
Frail	24	4.2	58.3	37.5	

^1^Including eyesight, hearing, dental, motion, speaking disorders

*Abbreviations*: *MMSE* mini-mental state examination, *ADL* Activities of Daily Living, *IADL* Instrumental Activities of Daily Living

**Fig. 1:**
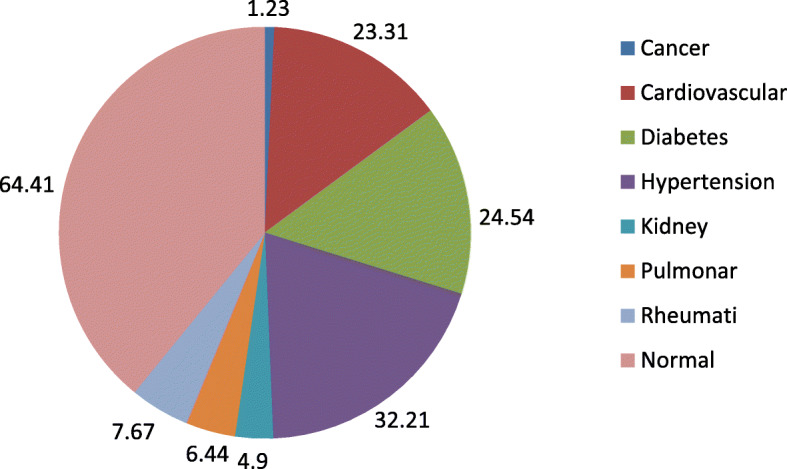
The percentage of major diseases associated with frailty

The prevalence of major diseases associated with frailty is shown in Fig. 1. According to the figure, hypertension, diabetes and cardiovascular disease were the most prevalent diseases. Frequency of poor nutrition among participants suffering from cancer, pulmonary disease, cardiovascular disease, diabetes and hypertension was significantly higher than normal subjects. Rheumatic, musculoskeletal and kidney diseases were not associated.

Table 3 shows multiple logistic regression analysis results. Model 1, depicts independent association among poor nutritional status and multiple personal characteristics include, age, gender, low educational status, financial dependency, inadequate family income, and living alone or with children (compared to living with spouse).

**Table 3:** Binary logistic regression models for malnourished/ at malnutrition risk % vs. normal- nourishment on personal and health characteristics

Measures	Model 1 OR (95% CI)	***P***	Model 2 OR (95% CI)	***P***	Model 3 OR (95% CI)	***P***
*Socio demographic indicators*						
**Age** (year)	1.141 (1.012–1.264)	0.003			1.119 (0.236–1.176)	0.326
**Children** (number)	1.307 (0.786–2.150)	0.311				
**Gender** (male vs female)	0.863 (0.544–1.324)	0.445				
**Marital status** (single vs married)	1.520 (1.205–2.224)	0.034			1.368 (0.538–1.714)	0.213
**Education**		0.003				0.313
Primary/middle school vs Illiterate	0.868 (0.564–1.076)	0.164			0.814 (0.564–1.345)	0.283
High school vs Illiterate	0.643 (0.334–0.867)	0.008			0.589 (0.378–1.410)	0.628
University vs Illiterate	0.346 (0.233–0.762)	0.003			0.416 (0.264–1.360)	0.071
**Financial dependency** (independent vs dependent)	0.748 (0.486–0.903)	0.001			0.625 (0.233–0.938)	0.010
**Family Income**		0.002				0.437
Inadequate vs Adequate	1.148 (1.034–1.956)	< 0.001			1.223 (0.628–1.719)	0.684
Almost enough vs Adequate	1.082 (0.854–2.231)	0.732			1.218 (0.769–1.648)	0.763
**Live with**		< 0.001				< 0.001
Alone vs Spouse	1.153 (1.021–2.063)	0.001			1.249 (1.105–2.620)	< 0.001
Children vs Spouse	2.156 (1.455–3.487)	< 0.001			1.611 (1.112–2.328)	< 0.001
*Health status*						
**Physical disabilities number**				< 0.001		< 0.001
One vs none			1.118 (0.124 ± 1.156)	0.641	1.213 (0.735 ± 2.248)	0.515
Two vs none			1.107 (0.923 ± 1.172)	0.387	1.183 (0.649 ± 2.543)	0.528
≥ Three vs none			2.320 (2.115 ± 3.151)	< 0.001	2.183 (1.246 ± 3.250)	< 0.001
**Daily drug intake number**			1.136 (0.668–1.319)	0.362		
**Self-rated healthy**				< 0.001		0.356
Not sure vs not healthy			0.825 (0.543–1.150)	0.186	0.821 (0.438–1.129)	0.424
Like others vs not healthy			0.712 (0.446–0.931)	0.017	0.642 (0.421–1.262)	0.231
Better than others vs not healthy			0.628 (0.333–0.928)	< 0.001	0.586 (0.159–1.322)	0.121
**Chronic disease number**			2.254 (1.346–3.540)	< 0.001	2.148 (1.167–2.879)	< 0.001
**Smoking status** (yes vs no)			1.437 (0.790–2.258)	0.132		
**MMSE** (normal cognition vs low cognition)			0.440 (0.231–0.892)	< 0.001	0.456 (0.293–0.934)	< 0.001
**ADL** (Independent vs Dependent)			0.576 (0.343–0.948)	< 0.001	0.536 (0.327–0.976)	< 0.001
**IADL**				< 0.001		< 0.001
Moderate vs Dependent			0.558 (0.352–0.876)	< 0.001	0.782 (0.352–0.879)	0.042
Independent vs Dependent			0.332 (0.215–0.748)	< 0.001	0.319 (0.194–0.856)	< 0.001
**Frailty**				< 0.001		< 0.001
Pre frail vs frail			0.735 (0.174–1.027)	0.057	0.698 (0.328–1.137)	0.079
Normal vs frail			0.320 (0.231–0.868)	< 0.001	0.253 (0.117–0.729)	< 0.001

1. model 1: logistic regression analysis between poor nutritional status and personal characteristics; 2. model 2: relationship between health status and impaired nutritional status; 3. model 3: adjusted for all the variables that were significantly associated on previous models

*Abbreviations*: *MMSE* mini-mental state examination, *ADL* Activities of Daily Living, *IADL* Instrumental Activities of Daily Living

In model 2 (health status), a relationship (*p* < 0.001) was identified among poor nutritional status and several physical disabilities and comorbidity, perception better than individual health, impaired cognitive function, dependency on ADL and IADL and being frail compared to normal. The strongest nutritional risk factors were frailty (OR: 0.253), dependency vs total independency in IADL (OR: 0.319) and normal cognition vs low cognition (OR: 0.456).

Adjusted association among impaired nutritional status and aforementioned associated covariates is depicted in the last model (model 3). The Cox & Snell R^2^ and Nagelkerke R^2^ were 0.312 and 0.421 respectively, which means that combination of introduced explanatory variables, accounts for 31–42% of poor nutritional status variance. It appeared that living alone or with children is also linked with poor nutritional status, with the odds ratio being 1.25–1.6 fold higher than that of life with spouse. Furthermore, 37.5% lower risk of malnutrition was associated with financial independency.

In terms of health variables, multiple physical disabilities and comorbidities were also found to be highly correlated with lower nutritional status (OR: 2.183, CI95%:1.246 ± 3.250; OR: 2.148, CI95%:1.167–2.879). Furthermore, elderly people with low cognition were 2.19 times more at risk of malnutrition or malnutrition. Being Independent for ADL was associated with 46.4% risk reduction of poor nutrition (OR: 0.536, CI95%:0.327–0.976). Odds ratio as well, decreased with increased independency in IADL, rising moderate to total independency (0.782 to 0.319). Eventually, a strong and consistent association was demonstrated between nutritional status and frailty; frailty increased the risk of impaired nutrition 3.95 fold compare to normal people. However, the relation between poor nutritional status and pre-frailty was not significant.

## Discussion

The present study used the full MNA tool to determine the prevalence of impaired nutritional status in elderly people of 60 years and above. This is one of the few studies to describe the prevalence of malnutrition and its associated socio-economic and geriatric factors in Iran. We found that 3.0% of the participants were suffering from malnutrition while 25.1% were at risk of malnutrition. The findings also revealed that single marital status (unmarried, divorced or widowed), financial dependency, multiple physical disabilities and comorbidity, cognitive decline, impaired ADL and IADL function, and frailty were independently associated with the MNA in the multiple model.

A systematic review of the prevalence of poor nutrition in different parts of Iran revealed that the prevalence of malnutrition or at-risk of malnutrition among the free-living elderly is between 3 and 10.6% and 32.7–46.7%, respectively. However, this rate was 3.2–53.6% and 38.7–68.8% among the elderly residents of nursing homes [7]. Our findings were close to studies from other countries including a family practice setting study from Italy showing a 25% malnutrition rate [3], and 28% among Turkish community-dwelling elderly [19]. However, this prevalence was much lower, in studies from Spain and Australia with 13.5 and 16% respectively [2, 20]. In addition to those studies, the prevalence of malnutrition was high in the elderly population of India, 32.5% [8], as well as among frail people, 56% [21] and patients with cognitive impairment, 35% [22]. The variation of malnutrition prevalence between these studies can be explained by the differing definitions of malnutrition, non-similar ages of the elderly in various settings such as private households, general practice, communities and institutions, and also the inclusion of individuals from urban or rural areas, subjects with cognitive impairments or frailty. Due to all these differences, the prevalence found would be difficult to compare.

In terms of socio-economic status, financial independence had a strong inverse association with malnutrition; even after adjusting the potential confounding factors. In a study conducted on elderly of rural Bangladesh, low income was highly correlated with poor nutrition [23]. Other researches also noted the greater risk of malnutrition among elderly living in poverty [1, 4]. This may be the result of decreased food availability, low consumption of nutritious food [10] and increased food insecurity [24]. The socio-economic conditions also influence dietary choices and eating patterns thereby affecting the nutritional status [8].

Living alone was associated with a risk of malnutrition/malnutrition in our study. This finding is inconsistent with some previous studies showing that people who live alone are not at greater risk of malnutrition [9, 25]. Although the precise mechanism by which marriage confers health benefits is unclear, studies have shown that married elders have better health and longer life [8]. This may be explained by low nutritious food consumed by people who live alone which reduces their social activities and increased sense of disability [26]. Damiao et al. showed the effect of social interaction on nutritional status among elderly people; therefore suggests eating should be a social occurrence [10]. A new finding from the present study indicated that living with offspring posed a higher nutritional risk for the elderly than living alone. Davis et al. [27] found that living with a spouse as the best, and living with people other than one’s spouse even worse than living alone in terms of nutrient intake. Losing a spouse through death or divorce can lead to grief, loneliness, loss of social support, less social participation, etc.; all of which may affect nutritional status.

The results of comprehensive geriatric evaluation in the present study showed that comorbidity, multiple physical disabilities, decreased cognition, impaired ADL and IADL function and frailty were associated factors of poor nutrition. Schilp et al. [28] evaluated the incidence of malnutrition in the elderly during a nine-year follow-up and found an association between two or more chronic diseases and the risk of malnutrition. In a study amongst the elderly in Denmark, the researchers found that there was a relationship between the risk of malnutrition and hospitalization frequency [29]. One of the most important malnutrition outcomes includes elevated nosocomial infection risk, particularly pneumonia [28], and falls and fractures risk [30]. Many diseases also occur as a result of decreased food intake and metabolic changes with negative influences on energy balance [31, 32]. This leads to a malicious loop between malnutrition and disease. Some of our initial study findings were also suggestive of the relationship between simultaneous presences of more than two physical disability involving eyesight, hearing, dental, motion and speech disorders and poor nutritional status. This relationship remained constant after all potential confounders were included in the final model.

Among these geriatric conditions, a decline in cognitive functioning is itself a risk factor for malnutrition [1]. In the present study, however, a strong association was found between MMSE score and malnutrition or risk of malnutrition. Cognitive impairment causes an inability to shop and prepare meals, and later with increased cognitive impairment, a person can forget to eat.

Even mild cognitive decrease can be associated with dietary changes. A study carried out by Kimura et al. [33] suggested higher risks of malnutrition among elderly people within early stages of alzheimer compared to cognitively healthy individuals. The relationship between these two conditions is complex. A causal relationship may exist and it is the faulty correlation among poor eating habits, nutritional status, and cognitive impairment. However, Maseda et al. [4] founds no association between MMSE and poor nutrition. This is maybe because the majority of the individuals attending the geriatrics centers were healthy and without any cognitive impairment (cognitive impairment was found only among 6.5% of the subjects). However, most studies have found that patients with cognitive impairment exhibit poor nutritional status [1, 8, 34]. Sanders et al. [35] stated that poor nutritional status was related to the severity of Alzheimer’s disease and anticipates its rapid progress. It is concluded that proper nutritional status can positively influence cognitive decline prognosis; this indicated the important role of nutritional assessment during early interventions.

In the present study, ADL and IADL dysfunction also showed a significant relationship with malnutrition. Similar results were found in many of the previously conducted studies on outpatient elderly clinics [8, 20, 21]. Nevertheless, Ulger et al. [19] failed to find any important relationship between poor nutritional status and ADL, whereas the relationship with IADL was significant. In contrast, Maseda et al. [2] did not identify IADL as a risk factor as a nutritional risk factor, but ADL proved the existence of such association. Meal preparation and eating disabilities can be a major cause of malnutrition compared to other IADL and ADL disabilities. Altered nutritional status was shown to be present, even before functional dependency begins to develop [36]. Therefore one possible explanation could be that low energy and especially low protein intake leads to a loss of muscle mass and strength, and subsequent loss of daily function [37]; however, it is also conceivable that a loss of function can lead to an inability to feed oneself adequately.

Frailty was found in 69.4% our study participants who were malnourished or at risk of malnutrition. Malnutrition prevalence was 4.5 folds higher in the elderly with frailty compared to healthy subjects. Nutritional status was closely associated with the degree of frailty, so that prevalence of poor nutrition diminished progressively in the pre-frail group and non-frail group. However, the relation between poor nutritional status and prefrailty was not significant. Our result is comparable to the Maseda study [4] which showed a significant relationship between frailty and MNA categories with the majority of frail individuals at malnutrition risk. Similar results were obtained by Kurkcu et al. [21] and Boulos et al. [38]. Frailty is a condition that is caused by progressive deterioration of multiple physiological systems due to aging, and is characterized by diminished response to low-level stress events. Many factors can contribute to the pathogenesis of frailty, and nutritional status appears to play a key role. Malnutrition contributes to the development of frailty by accelerating the onset of sarcopenia [23].

Regardless of whether physical frailty or malnutrition occurs first, there is possibly a closed-loop cyclical association between the two in progression towards a combined frailty-malnutrition state. This association is reflected by the progressive increase in the prevalence of frailty across MNA-SF normal nutrition and at risk and malnourished groups, as well as the progressive increase in MNA-SF malnutrition prevalence from robust to prefrail to frail groups. However, this finding should be further investigated in prospective follow-up studies. The results of a study by Valentini et al. [22] indicated that, in the absence of physical frailty, poor nutrition was associated with only a small non-significant increase in adverse functional outcomes and mortality, while physical frailty was associated with a relatively greater increase the risks of poor function in the absence of poor nutrition. In contrast, a significant increase in adverse health outcomes was associated with co-occurrence of poor nutrition and physical frailty.

It is widely believed that extensive physiological and psychosocial changes due to aging would make it more difficult for the elderly to meet their nutritional needs. However, those elderlies who suffer from geriatric problems such as physical dysfunction, cognitive decline and frailty, would face difficulty meeting their nutritional needs anyways. Meeting the nutritional needs of these people is very important for maintaining their health, functional independence and quality of life. In Iran, health treatment system and policies aim to make changes in the health care system in favor of the society; in its new policies, it is important to consider the nutritional needs of the elderly, especially those affected by geriatric problems. This is likely leads to an increase in independence and reduction in subsequent requests for social care and hospital admissions which leads to a less invasive and expensive health care.

Incorporating multiple evaluation sets of potential socio-demographic and health determinants is an important strength of this study. These determinants have been analyzed to determine their possible association with presence or risk of malnutrition. However, this study includes several limitations, such as the cross sectional design by which establishing causality isn’t possible. In addition, information bias may be present because of cognitive status differences among individuals. Another limitation of this study is reliance on self-report for some measures including type and number of chronic diseases. Also, some remaining unidentified factors and residual bias may be present. Finally, we used non-probabilistic sampling, in which the samples might did not represent the actual population, properly.

## Conclusion

From this study, we conclude that elderly individuals who suffer from multiple physical disabilities, chronic illnesses and cognitive and functional decline, those who are financially poor, and those with a single marital status were at higher risk of malnutrition. Impaired nutritional status was clearly related to frailty. To clarify the findings, more research is needed. In addition to that, raising awareness of health professionals and caregivers about malnutrition needs to be addressed in the context of the demographic changes. Special training is required to strengthen the community-based knowledge about nutrition and to provide routine screening for vulnerable groups such as elderly with financial hardships, those without a spouse or those suffering from multiple diseases. Similarly, elderly people have to be provided with newly developed nutritional guidelines.

## Data Availability

The datasets obtained and/or analysed during the current study are not publicly available as the datasets are highly detailed and we are planning to publish more papers using the same dataset.

## Abbreviations

MNA: Mini nutritional assessment
MMSE: Mini-mental state examination
ADL: Activities of Daily Living
IADL: Instrumental Activities of Daily Living Scale
